# Non-Repetitive Scanning LiDAR Sensor for Robust 3D Point Cloud Registration in Localization and Mapping Applications

**DOI:** 10.3390/s24020378

**Published:** 2024-01-08

**Authors:** Ahmad K. Aijazi, Paul Checchin

**Affiliations:** Université Clermont Auvergne, Clermont Auvergne INP, CNRS, Institut Pascal, F-63000 Clermont-Ferrand, France; paul.checchin@uca.fr

**Keywords:** LiDAR, Spirograph scanning pattern, non-repetitive scan, 3D point cloud and scan registration

## Abstract

Three-dimensional point cloud registration is a fundamental task for localization and mapping in autonomous navigation applications. Over the years, registration algorithms have evolved; nevertheless, several challenges still remain. Recently, non-repetitive scanning LiDAR sensors have emerged as a promising 3D data acquisition tool. However, the feasibility of this type of sensor to leverage robust point cloud registration still needs to be ascertained. In this paper, we explore the feasibility of one such LiDAR sensor with a Spirograph-type non-repetitive scanning pattern for robust 3D point cloud registration. We first characterize the data of this unique sensor; then, utilizing these results, we propose a new 3D point cloud registration method that exploits the unique scanning pattern of the sensor to register successive 3D scans. The characteristic equations of the unique scanning pattern, determined during the characterization phase, are used to reconstruct a perfect scan at the target distance. The real scan is then compared with this reconstructed scan to extract objects in the scene. The displacement of these extracted objects with respect to the center of the unique scanning pattern is compared in successive scans to determine the transformations that are then used to register these scans. The proposed method is evaluated on two real and different datasets and compared with other state-of-the-art registration methods. After analysis, the performance (localization and mapping results) of the proposed method is further improved by adding constraints like loop closure and employing a Curve Fitting Derivative Filter (CFDT) to better estimate the trajectory. The results clearly demonstrate the suitability of the sensor for such applications. The proposed method is found to be comparable with other methods in terms of accuracy but surpasses them in performance in terms of processing time.

## 1. Introduction

The advancement of LiDAR (Light Detection And Ranging) technology has revolutionized the field of three-dimensional (3D) sensing and mapping, playing a pivotal role in various applications such as autonomous vehicles, robotics and environmental monitoring. Registering 3D point clouds obtained from these LiDARs is a fundamental task for localization and mapping in autonomous navigation. Registration algorithms have evolved over the years; however, several challenges still remain.

Some of the critical issues include accuracy, noise reduction, robustness, scalability and real-time processing. Most of the state-of-the-art methods employ traditional LiDAR sensors with repetitive scanning patterns, which have inherent limitations. Recently, non-repetitive scanning LiDAR sensors have emerged as a promising 3D data acquisition tool. However, the feasibility of this type of sensor to leverage robust point cloud registration still needs to be ascertained. In this paper, we explore the feasibility of one such LiDAR sensor with a Spirograph-type non-repetitive scanning pattern for robust 3D point cloud registration.

The work presented in this paper represents a promising advancement toward more robust and accurate 3D point cloud registration, with implications for the continued evolution of technologies reliant on LiDAR-based environmental sensing.

## 2. Related Work

Point cloud registration is a fundamental task in computer vision and robotics, enabling the alignment of multiple 3D scans to create a coherent and comprehensive model of a scene or object. Over the years, numerous techniques and algorithms have been developed to address this challenging problem. In this section, we review some key research in the field of 3D point cloud registration.

The Iterative Closest Point algorithm (ICP) proposed by [1] is a foundational method in point cloud registration. ICP iteratively minimizes the distance between corresponding points in two point clouds, allowing for rigid transformation estimation. Extensions such as the Generalized ICP (GICP) by [2], dense normal-based ICP (NICP) [3] and Sample Consensus ICP [4] have improved its robustness and applicability to various scenarios.

Tazir et al. in [5] modified the method for selecting 3D points for the matching process in the standard ICP algorithm to propose Cluster Iterative Closest Point (CICP), successfully registers point clouds of different densities obtained from different sensors. Vizzo et al. in [6] presented KISS-ICP, which relies on point-to-point ICP combined with adaptive thresholding for correspondence matching, a robust kernel and a point cloud subsampling strategy. This resulting system requires only a few parameters that in most cases do not need to be tuned for different LiDAR sensors or a changing environment. Another variant, Global ICP (G-ICP), presented by Aijazi et al. in [7], employs bundle adjustment to fine tune the registration process at the end, in addition to scan-by-scan registration using point-to-plane matching; however, such a method is not suitable for real-time processing.

Feature-based registration methods focus on extracting distinctive features from point clouds and matching them to establish correspondences. The SIFT-based approach [8], Fast Point Feature Histograms (FPFH) [9] and Signature of Histogram of Orientations (SHOT) [10] are notable examples. Ghorbani et al. [11] evaluated the use of 3D keypoint detectors like the 3D SIFT and SHOT method to register sequential indoor and outdoor point clouds. The authors identified quantity, quality and spatial distribution of the extracted key points along with the matching of extracted features as the main challenges in 3D point cloud registration using these approaches.

While rigid registration is suitable for aligning objects with minimal deformation, non-rigid registration methods are essential for deformable object alignment and medical image registration. The Coherent Point Drift (CPD) algorithm by Myronenko and Song [12] is a significant advancement in this domain.

Scalability is a critical concern for point cloud registration, especially in applications like autonomous driving and mapping. A method to improve the robustness of scale estimation is the use of a smooth variable structure filter (SVSF) in SLAM applications, i.e., SLAM-SVSF [13]. The LeGO-LOAM system by [14] offers a real-time solution for large-scale point cloud registration and mapping. The method is an extension of Lidar odometry and mapping (LOAM) presented by [15] that computes the odometry by registering planar and edge features to a sparse feature map. Lego-LOAM added ground constraints to improve accuracy; more recently, F-LOAM [16] modified the original method to make it faster by using a more efficient optimization technique. However, these methods rely on several predefined parameters that need to be tuned depending on sensor resolution, environment structure, etc.

A Surfel-based method SuMa [17] was proposed for LiDAR odometry estimation and point cloud registration. The method was extended to incorporate semantics [18] and handle dynamic objects [19]. Deschaud [20] introduced the implicit moving least square surface (IMLS-SLAM) [21] while Vizzo et al. [22] exploited a triangular mesh as the internal map representation. Both these methods rely on a point-to-plane metric to register consecutive scans. However, this requires the estimation of surface normals as additional data-dependent parameters.

Recent advances in deep learning have led to the emergence of deep neural networks for point cloud registration [23,24]. However, applying deep learning to 3D point cloud registration presents several challenges due to the irregular (i.e., the points are not evenly distributed spatially across the different regions of the scene), unstructured (i.e., usually not organized in a known pattern) and unordered (i.e., mostly the point cloud of a scene is stored as a set of points where changing the order does not change the scene representation) nature of the 3D LiDAR data. These issues make the direct application of convolutional neural networks (CNNs) difficult, as they assume ordered and regular structures.

With the recent advent of non-repetitive scanning LiDARs, some work has also been conducted to register their point clouds. As an extension of classical feature-based methods, the work by [25] introduced a feature-based registration algorithm employing point clouds generated by non-repetitive scanning LiDARs, leveraging distinct features to establish correspondences. Wang et al. [26] proposed a novel method that leverages deep learning techniques for sparse point cloud registration, achieving impressive results in various scenarios. He et al. [27] introduced a highly efficient algorithm designed for non-repetitive scanning LiDARs, combining geometric and learning-based approaches to improve speed and accuracy. Li et al. [28] presented a registration method for combining non-repetitive scanning LiDAR point clouds with RGB-D data, enabling precise 3D scene reconstruction. Zou et al. [29] presented a plane-based global registration (PGR) approach for coarse registration followed by an ICP algorithm to register point clouds.

In this paper, we first present comprehensive characterization results of such a non-repetitive LiDAR sensor. Different aspects of this new type of sensor are studied for its effective utilization for localization and mapping applications applied to Autonomous Ground Vehicles (AGV). In order to demonstrate the feasibility of this sensor, we use these results to propose a new 3D point cloud registration method—for localization and mapping—that exploits the unique scanning pattern of the sensor to register successive 3D scans. To the best of our knowledge, no work has so far exploited the non-repetitive scanning pattern to effectively register 3D point clouds.

In the following sections, we first introduce the new LiDAR sensor and present the comprehensive characterization results (Section 3). In order to demonstrate its feasibility for localization and mapping applications, we then propose a new 3D registration method (Section 4) that exploits the unique scanning pattern of the sensor. The experiments and results, as well as the discussions, are presented in Section 5 and Section 6; then, we conclude in Section 7.

## 3. Characterization of Spirograph Scanning LiDAR

The recently developed MID-70 sensor by LIVOX [30] is a solid-state 3D LiDAR. The sensor is capable of collecting up to 100,000 points per second within a circular field of view directly in front of the scanner’s glass window. With a small form factor, it has a range of about 260m with a precision of up to 0.02m (see Table 1 for more specifications). However, what really differentiates this sensor from other laser scanners is its scanning mechanism. Rather than rotating the laser scanner/receiver pair themselves (as is done in the case of multi-line scanners like Velodyne LiDARs), it holds the two in a “solid state”, fixed inside the scanner’s housing, while the outbound and inbound light are directed through a pair of Risley prisms [31]. These wedge-shaped glass lenses act as optical steering mechanisms as they rotate in opposite directions, directing the beam in a Spirograph scanning pattern in the circular scan area.

In order to effectively use such a sensor, the characterization of its data is indispensable, especially given that, being a new sensor, few performance data are available. In order to characterize the sensor data, different tests were conducted, and the results are presented in the following sections.

### 3.1. Startup Time

Once the sensor is powered up, it does not start immediately. There is a slight delay (≈8 s) for safety, to ensure the smooth functioning of the sensor and to prevent any erroneous data upfront. Each scan contains about 100,000 valid points/s.

### 3.2. Range and Field of View (FOV)

**Figure 1:**
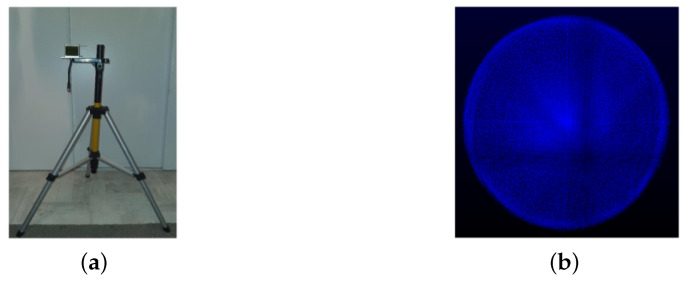
(**a**) Sensor mounted on the tripod. (**b**) Circular point cloud of a flat wall.

**Figure 2:**
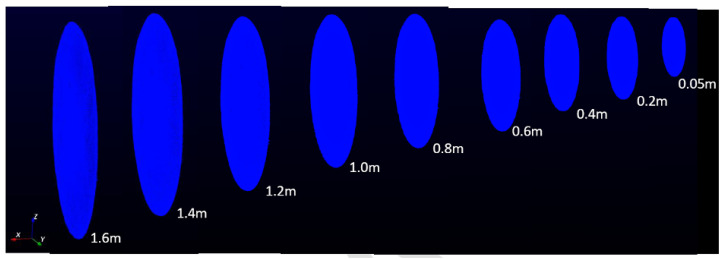
Point clouds from sensor at close range (1.6m to 0.05m).

**Figure 3:**
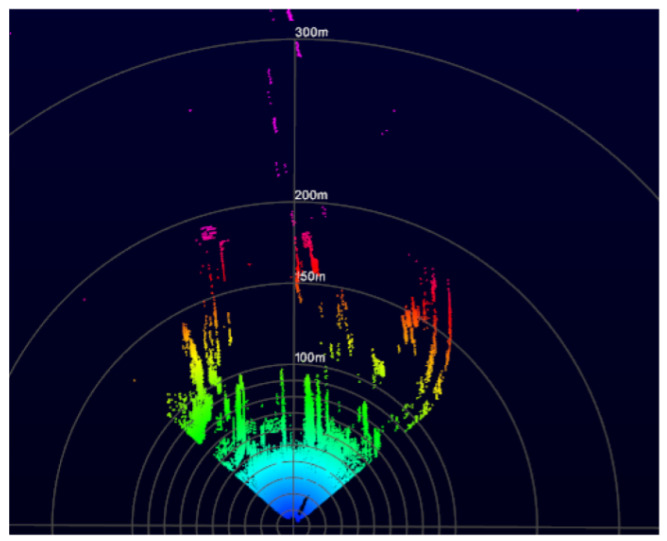
Sensor in outside environment to determine maximum range.

The sensor was mounted on a tripod, as shown in Figure 1a, and tested in both inside and outside environments with both close and far objects detected in the scene. The objective was to detect the closest and farthest object to determine the minimum and maximum range of the sensor. The minimum and maximum range were found to be 0.05m (see Figure 2) and more than 200m, respectively (see Figure 3). However, for close range between 0.05m and 0.25m, the accuracy is slightly degraded, mainly because of the filtering applied in the sensor system.

In order to measure the Field Of View (FOV), the sensor was mounted on a tripod and placed, parallel, at a fixed distance in front of a flat wall. The captured point clouds were studied and, using basic trigonometry, the effective FOV was determined to be almost circular (average difference between vertical and horizontal diameter < 1.1%), as shown in Figure 1b.

### 3.3. Drift Analysis

**Figure 4:**
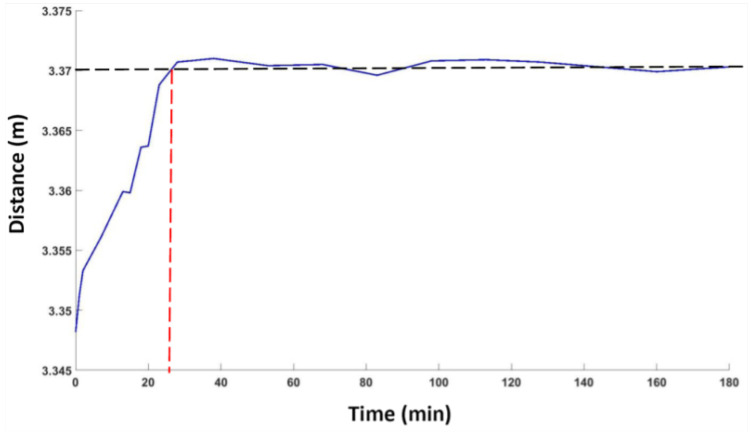
Drift analysis showing the variation in distance measurement with respect to time.

The drift analysis gives an idea about the stability of the LiDAR sensor. In order to analyze this effect of the 3D LiDAR sensor, measurements of a plane surface were performed over a long period of time. The LiDAR sensor was placed at a fixed distance from a white wall and the z-axis of the sensor was kept vertical to the plane of the wall. Repeated measurements were taken over a period of 3 h. The reference distance (ground truth) for all experiments was measured using Leica’s Disto D110 distance measurement unit [32], with an accuracy of ±1.5mm. The unit was carefully placed at the same spot (marked by the manufacturer) on the sensor that corresponds to the sensor measurement zero. The average of multiple readings was taken, at each instance, to minimize the effect of noise and further improve measurement accuracy. Figure 4 shows the variation in distance measurements with respect to time. A large variation of about 2 cm was observed in the first 24 min of running; then, the measurement value stabilized. As the drift effect is mainly due to the variation in temperature of the device during functioning, the results indicate that the sensor achieved its ambient working temperature by this time.

### 3.4. Effect of Surface Colors

**Figure 5:**
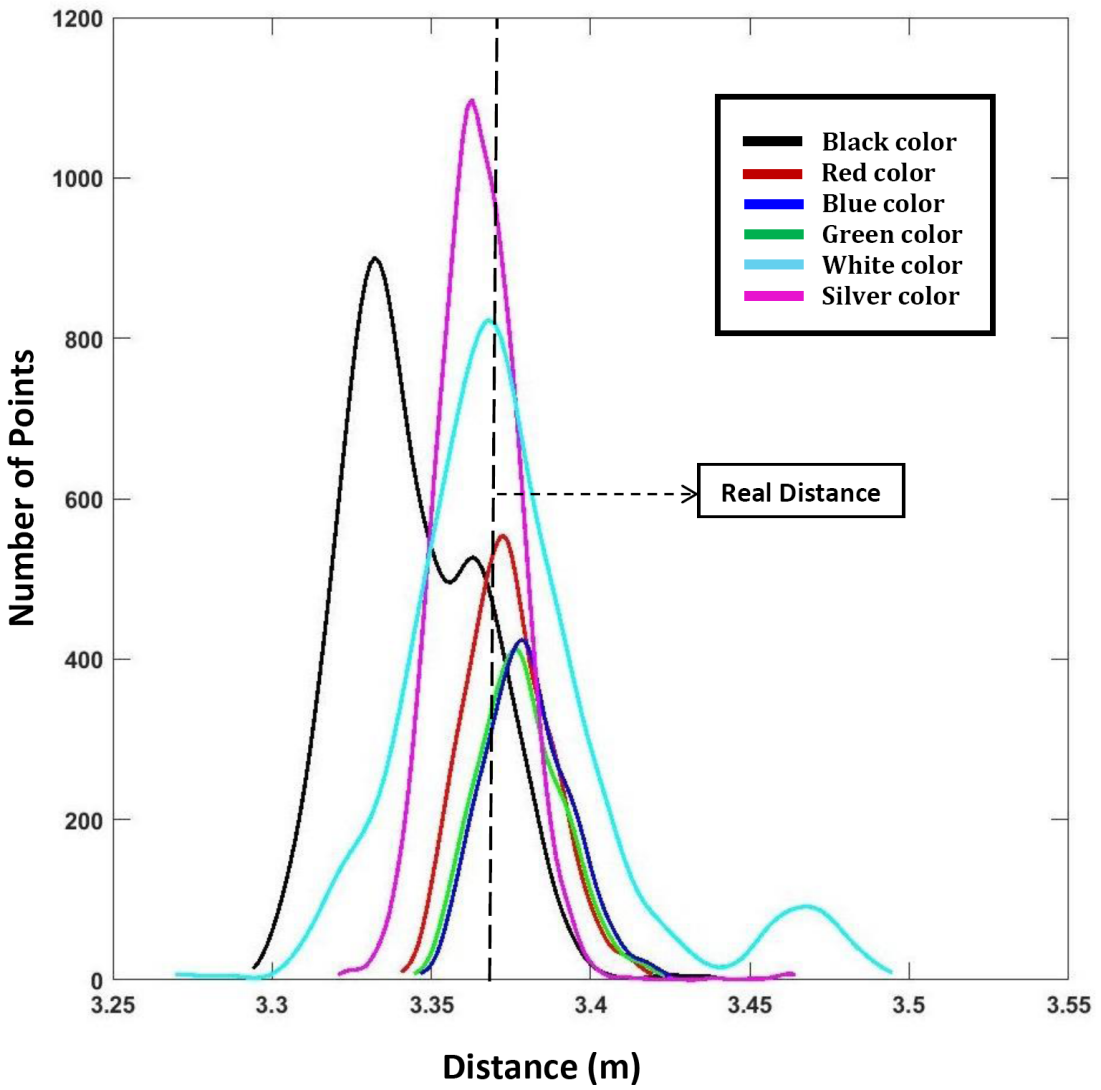
Variation in distance measurement with respect to different colors.

The color of the target’s surface affects the laser measurements. In order to evaluate the LiDAR’s performance against different colors, the LiDAR was tested on three primary colors—red, green and blue; two secondary colors—black and white; and also a shiny silver (high reflectivity) color. Each of these 6 different colored targets are made up of the same material—i.e., paper—and were all fixed on exactly the same place during testing. The real/reference distance was measured in the same manner as mentioned in Section 3.3. Figure 5 presents the results in the form of distance distributions.

### 3.5. Effect of Surface Material

**Figure 6:**
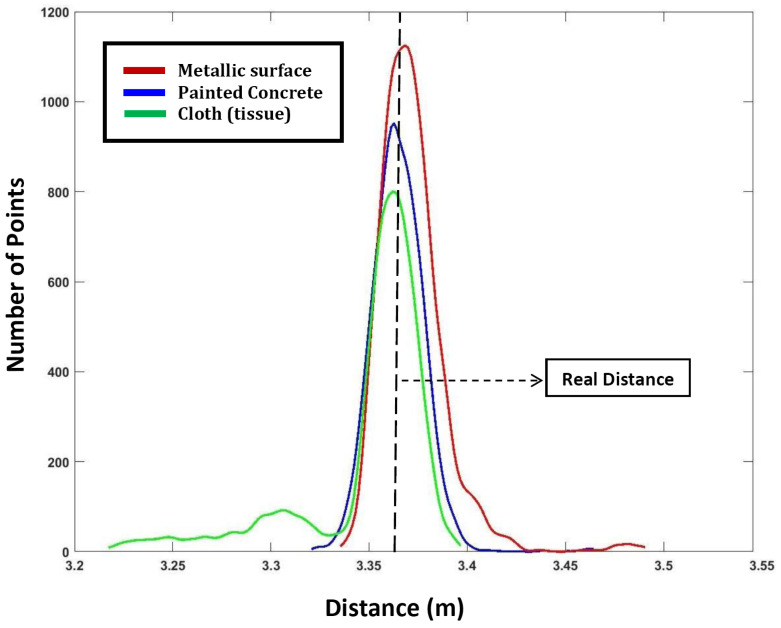
Variation in distance measurement with respect to different materials.

Just like the surface color, the material of the surface also affects the reflection of the laser. In order to evaluate this effect, tests were conducted on three different target materials. They were all white in color; however, the materials were different: tissue cloth, concrete and metal. The real/reference distance was measured in the same manner as mentioned in Section 3.3. The results are shown in Figure 6.

Even though it could be seen that the variation in distance measurements due to the different materials was not much, the number of reflected 3D points was much higher for the metallic surface compared with that of the other two.

### 3.6. Influence of Luminosity (Ambient Light)

**Figure 7:**
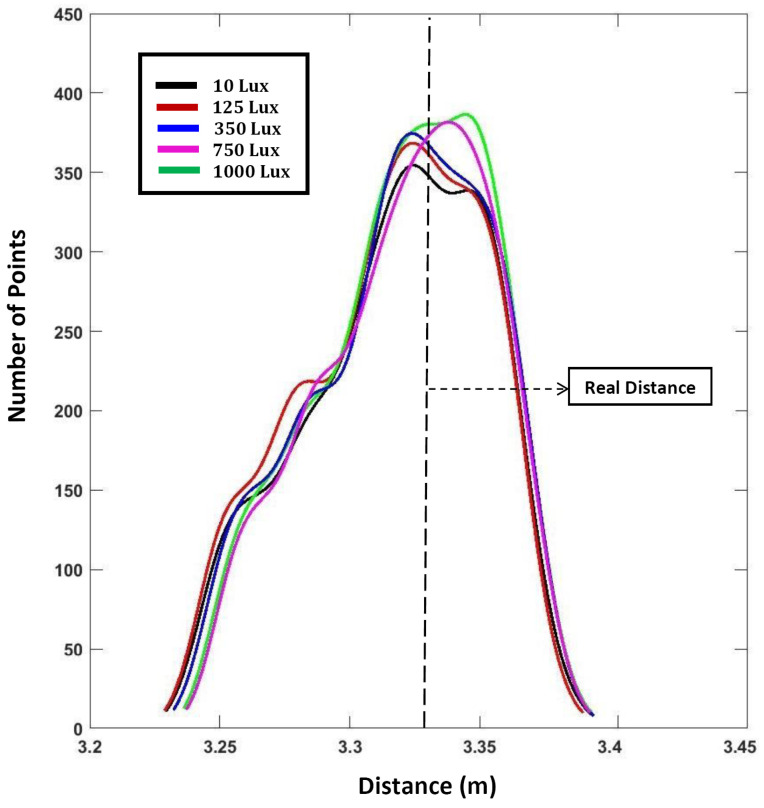
Variation in distance measurement with respect to different luminosity levels.

The sensor’s performance at different luminosity levels was evaluated by measuring the distance of a fixed white target. The real/reference distance was measured in the same manner as mentioned in Section 3.3. The luminosity levels were modified using a large external lamp. A LUX meter was used to measure the luminosity levels. The results presented in Figure 7 show that there is not much variation in distance measurements, implying that the sensor is quite robust to changes in luminosity.

### 3.7. Problem of Pixel Mixing

When a laser beam falls at the very edge of a surface, the measured range is taken as a combination of the foreground and background surfaces/objects and lies in between the two distances. This condition is called “mixed pixels” [33].

**Figure 8:**
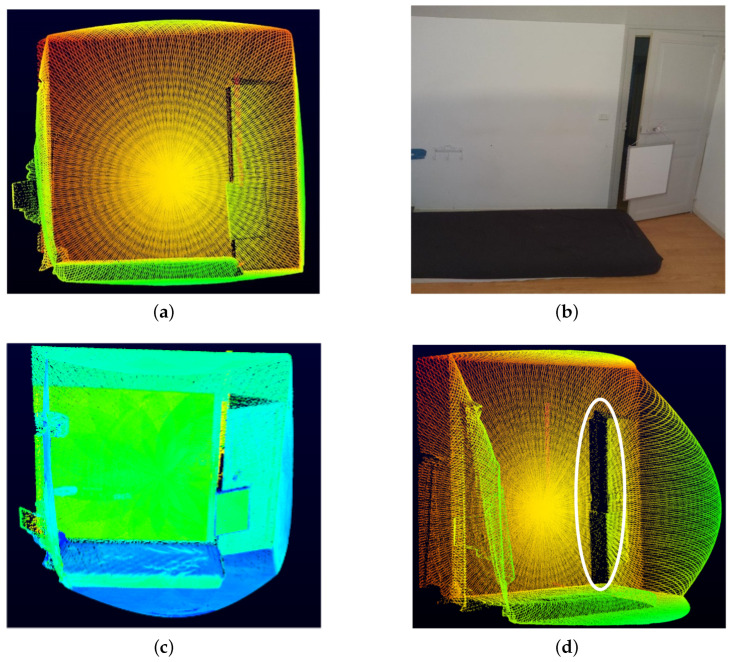
Study of the phenomena of pixel mixing. In (**a**), we find the original point cloud while in (**b**) and (**c**) the RGB and intensity images are presented. (**d**) Circled in white, we find a few false points due to the problem of mixed pixels.

In order to evaluate this phenomenon, we scanned a white wall with a slightly opened door, as shown in Figure 8. The sensor was placed at a distance of 4.5m. The aim was to analyze the mixed pixels, also called jump edges, at the door opening.

The number of false measurements (points) due to pixel mixing was found to be very few, as shown in Figure 8d (encircled in white). These can be easily removed or filtered out in post-processing, as presented in [34,35].

### 3.8. Distance Analysis

**Figure 9:**
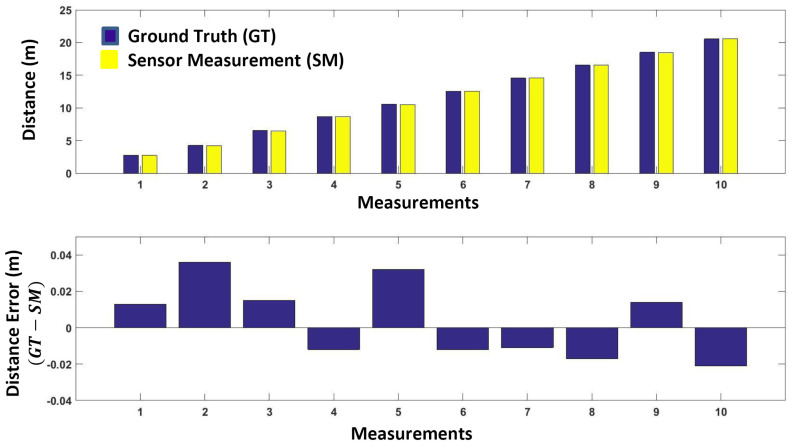
Measurement accuracy with respect to distance (range).

In order to determine the effect of target distance, we took the measurements of a fixed target surface at different distances (1m to 10m). The real/reference distance (ground truth) was measured in the same manner as mentioned in Section 3.3. The measured distances with respect to the ground truth are presented in Figure 9. A small measurement error with a standard deviation of 2.08cm indicates the sensor’s high precision.

### 3.9. Scanning Pattern

**Figure 10:**
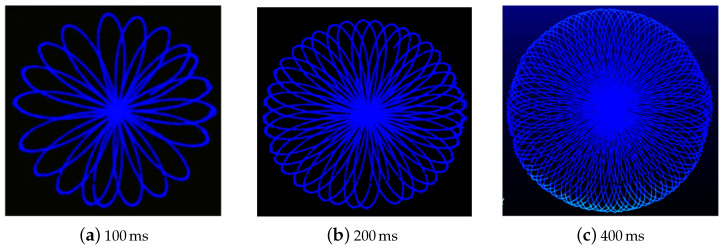
Rosette-style scanning pattern of the sensor scans at different integration times.

In order to determine the scan pattern of the LiDAR sensor, its scans were analyzed (of a plane surface) at different integration times (resolution) and distances, as shown in Figure 10. The non-repetitive Spirograph-type pattern was determined to be a rosette.

A rosette is a set of points in polar coordinates (r,θ) specified by the following polar equation:

(1) r=acos(kθ),

or in Cartesian coordinates (x,y) using the following parametric equations:

(2) x=rcos(θ)=acos(kθ)cos(θ),

(3) y=rsin(θ)=acos(kθ)sin(θ),

where *a* is the amplitude or size factor and *k* is the angular frequency. The rose specified by r=asin(kθ) is identical to that specified by r=acos(kθ) rotated counter-clockwise by $\frac{\pi }{2K}$ radians, which is one-quarter the period of either sinusoidal. If *k* is odd, the rosette is *k* petaled. If *k* is even, the rosette is 2k petaled.

**Figure 11:**
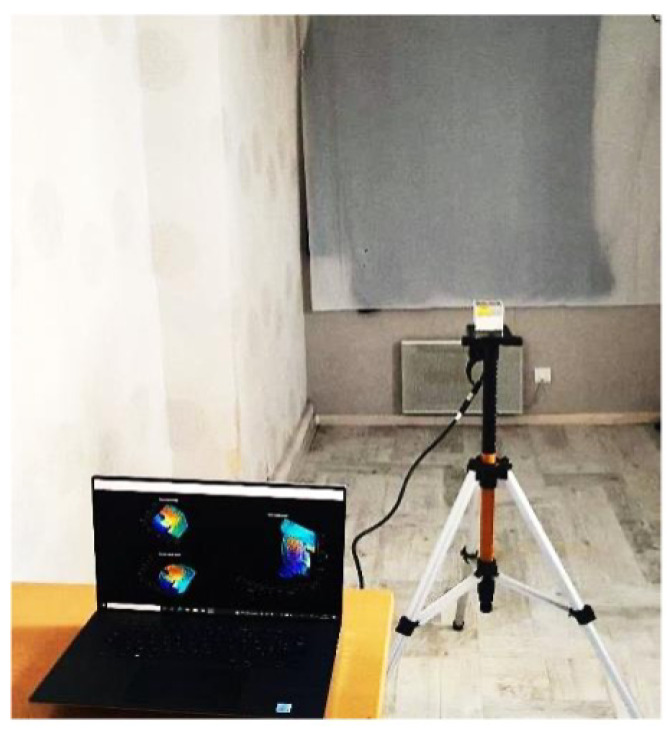
Experimental setup.

In order to exploit the scanning pattern of the LiDAR sensor, we need to characterize its scanning pattern by determining the value of *a* and *k*. In order to determine these parameters, we positioned the sensor on a tripod stand in front of a plane surface, as shown in Figure 11.

Concerning the value of *k*, the different scans obtained from the sensor were analyzed (the form and number of petals) at different integration times, as shown in Figure 10. The number of petals found was 200 per second. Being an even number, this equates to a value of k=100.

**Figure 12:**
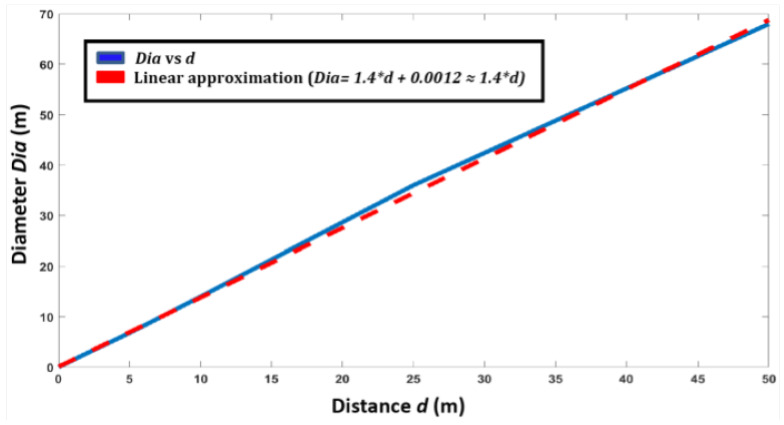
The size (in terms of diameter) of the rosette scan with respect to the target distance.

We considered the value of *a* as a function of scanning distance. The distance of sensor was varied from 1 to 50m at regular intervals, and the series of scan patterns obtained was studied (cf. Section 3.3). Figure 12 presents the size (in terms of diameter) of the rosette scan in terms of distance.

Using a linear approximation, the value of *a* was found as follows:

(4) a=1.4×d,

where *d* is the target distance.

The characterization results presented in this section show the aptness of the sensor for localization and mapping applications. The sensor’s performance remained mostly invariant to changes in target characteristics and environment. In order to further demonstrate the suitability of the sensor for such applications, we present, in the next section, a new 3D point cloud registration method that exploits the unique characteristics of the sensor.

## 4. Three-Dimensional Scan Registration

**Figure 13:**
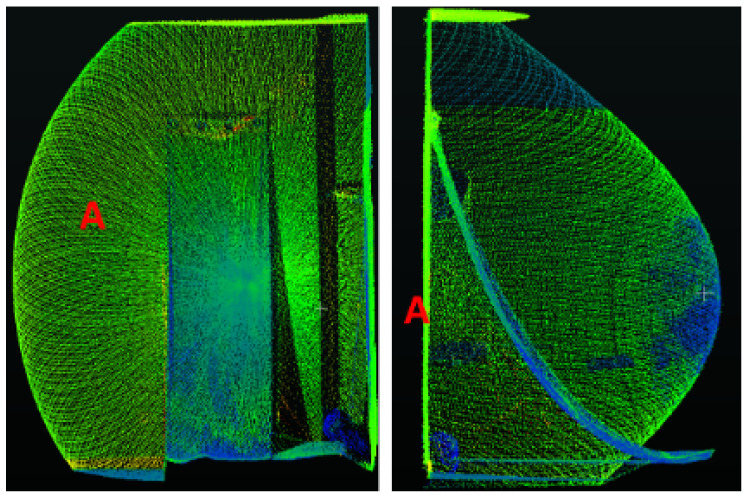
The distance to the farthest vertical plane (A), perpendicular to the sensor plane, is considered as *d*.

The scans are registered one by one in succession. In order to register two successive scans $s_{\;i}^{\;o}$ and $s_{\;j}^{\;o}$, where *i* and *j* are the scan numbers, we first reconstruct an ideal/perfect Rosette-style scan (without deformations due to objects) at the specified target distance *d*, applying the Equations (2) and (3) and using the values of *k* and *a* determined in the previous section. The distance *d* in (4) is taken as the distance of the farthest vertical plane, perpendicular to the sensor plane, detected in each scan, as shown in Figure 13.

**Figure 14:**
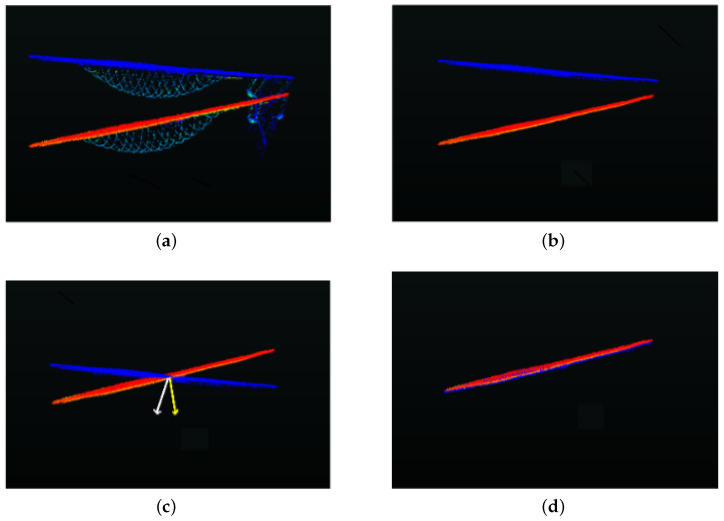
(**a**) The two successive scans with objects, in red and blue respectively. (**b**) The reconstructed scans corresponding to the two original scans without objects. In (**c**), the centers of the two scans are aligned and normal vectors are estimated, while in (**d**), the second scan is rotated to align with the first scan and the difference of normal vectors give $\theta_{x},\theta_{y},\theta_{z}$.

The centers $c_{\;i}^{\;r}$ and $c_{\;j}^{\;r}$ of the successive reconstructed Rosette-style scans $s_{\;i}^{\;r}$ and $s_{\;j}^{\;r}$ are first aligned; then, in the second step, their orientations are aligned and rotations around the three axes—i.e., $\theta_{x}$, $\theta_{y}$ and $\theta_{z}$, respectively—are estimated, as shown in Figure 14.

In order to align the orientation, the surface normal vector is estimated for each of the reconstructed scans using Principal Component Analysis (PCA) [36]. Given a set of points in each reconstructed scan $D={x_{\;i}}_{\left(i=1\right)}^{np}$, the PCA surface normal approximation for a given data point p∈D is typically computed by first determining the K-Nearest Neighbors, $x_{K}\in D$, of *p*. Given the *K* neighbors, the approximate surface normal is then the eigenvector associated with the smallest eigenvalue of the symmetric positive semi-definite matrix

(5) $$V=\sum_{k=1}^{K}{\left(x_{k}-\bar{p}\right)}^{T}\left(x_{k}-\bar{p}\right)$$

where $\bar{p}$ corresponds to the centers $c_{\;i}^{\;r}$ and $c_{\;j}^{\;r}$ of the successive reconstructed Rosette-style scans $s_{\;i}^{\;r}$ and $s_{\;j}^{\;r}$.

The rotation around the three axes is then estimated as the difference between the orientation of the two normal vectors. As the sensor remains on the ground, the rotation in successive scans around the axis perpendicular to the sensor plane is considered zero.

(6) $$\begin{array}{l} \theta_{x}=(\theta_{x1}-\theta_{x2})\\ \theta_{y}=(\theta_{y1}-\theta_{y2})\\ \theta_{z}=(\theta_{z1}-\theta_{z2})\approx 0 \end{array}$$

where $\theta_{x1}$, $\theta_{y1}$, $\theta_{z1}$ and $\theta_{x2}$, $\theta_{y2}$, $\theta_{z2}$ are the orientations of the two vectors with respect to the *x*, *y* and *z* axes, respectively.

**Figure 15:**
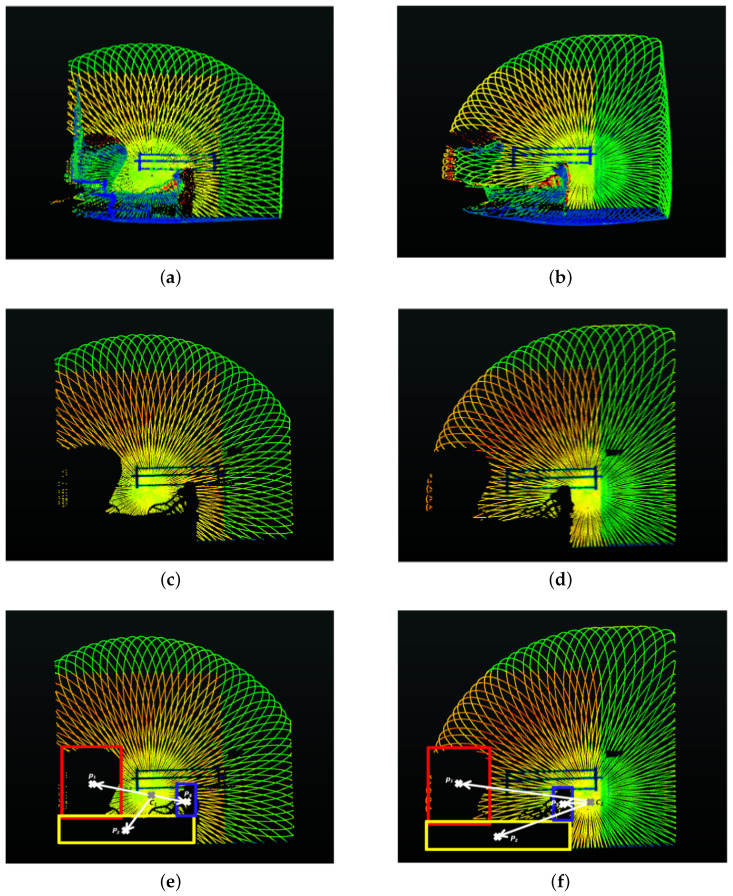
(**a**,**b**) The two original successive scans with objects. (**c**,**d**) The two scans with holes after extraction of objects. (**e**,**f**) The bounding box around the different holes in both scans, along with their centroids and the vectors from the center of the scans and the centroids. The difference in these vectors (displacement of objects with respect to the scan center) provides us the estimates of $t_{x}$ and $t_{y}$.

The original scans $s_{\;i}^{\;o}$ and $s_{\;j}^{\;o}$ are then matched with the corresponding reconstructed scans $s_{\;i}^{\;r}$ and $s_{\;j}^{\;r}$. As the reconstructed scans are free of objects, this allows the extraction of objects present in the original scans, leaving behind holes. The reconstructed scans help complete the farthest and largest vertical plane in the original scans occluded by objects in the scene. All 3D points lying outside this vertical plane are then considered as object points and are extracted, leaving behind holes. The centroid *P* of the resulting holes in the original scans are calculated using a bounding box around each hole (or extracted object) with respect to $c_{\;i}^{\;o}$ and $c_{\;j}^{\;o}$, as shown in Figure 15. The displacement of these centroids with respect to the center of the scans in the successive scans provides us with the translation, i.e., $t_{x,y}$, between these two scans:

(7) $$t_{x,y}=\frac{1}{n}\sum_{m=1}^{n}\left(\left(P_{\;j}^{\;m}-c_{\;j}^{\;o}\right)-\left(P_{\;i}^{\;m}-c_{\;i}^{\;o}\right)\right)$$

where *n* is the total number of holes (due to extracted objects) in the scans, and $P_{\;i}^{\;m}$ and $P_{\;j}^{\;m}$ are the centroids of the $m^{\mathrm{th}}$ hole in the scans $s_{\;i}^{\;o}$ and $s_{\;j}^{\;o}$, respectively. The displacement along the *z* axis is denoted $t_{z}=\left(d_{\;i}-d_{\;j}\right)$, where $d_{\;i}$ and $d_{\;j}$ are the distances of the farthest vertical plane, perpendicular to the sensor plane, detected in each scan, respectively.

These rotations $R(\theta_{x},\theta_{y},\theta_{z})$ and translations $T(t_{x},t_{y},t_{z})$ are then used to transform the original scans, with the first scan taken as reference, using (8). The transformed scan and the reference scan are then aggregated (9) to form the new reference scan for the next scan.

(8) $${\hat{s}}_{\;j}^{\;o}=R.s_{\;j}^{\;o}+T$$

(9) $$s_{\;i+1}^{\;o}={\hat{s}}_{\;j}^{\;o}+s_{\;i}^{\;o}$$

The method is summarized in Algorithm 1.
**Algorithm 1:** Three-Dimensional Scan Registration.
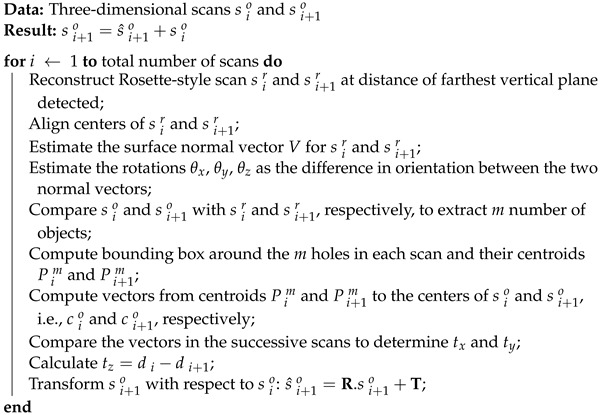


## 5. Experimentation Setup

In order to evaluate the proposed method, the sensor was employed in both static and dynamic modes.

### 5.1. Static Mode

**Figure 16:**
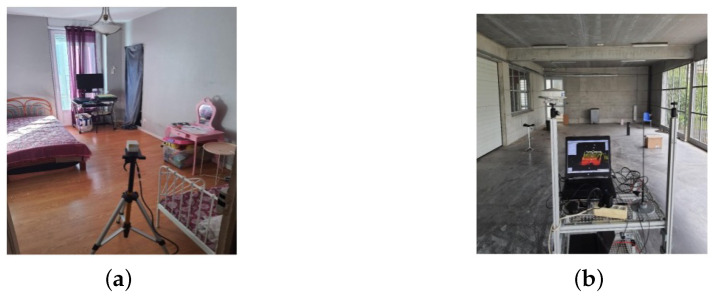
(**a**,**b**) The experimental setup for experiments Exp_1 and Exp_2.

In this mode, the sensor was mounted on a tripod and moved around different positions to completely scan an indoor room equipped with furniture, as shown in Figure 16a. The scanning was performed at an integration time of 0.2 s. This experiment Exp_1 contained a total of 35 scans.

### 5.2. Dynamic Mode

In this mode, the LiDAR sensor was mounted on a small mobile robot moving on a predefined trajectory, at a speed of about 1m/s, scanning an enclosed hangar/parking lot, as shown in Figure 16b. The robot was also equipped with a 2D camera, an odometer and low-cost MEMS-based (Microelectromechanical systems) IMU (Inertial Measurement Unit). Scanning was performed at an integration time of 0.2 s. Exp_2 contains 357 scans.

In both experiments, loop closure was ensured to help in the evaluation process, while the ground truth was obtained via physical measurements of the different room dimensions. These dimensions were measured using Leica’s Disto D110 distance measurement unit, having an accuracy of ±1.5mm. The average of multiple readings was taken, for each dimension, to minimize the effect of noise and further improve measurement accuracy. These dimensions include windows and door openings, the length and height of all the walls and the length and width of the floor. All LiDAR measurements were taken after 30min (>24 min) of running to obtain more accurate results, as concluded in Section 3.3; as a precautionary measure, all measurements less than 1m were discarded (Section 3.2).

## 6. Results and Discussion

Using the data obtained from the two experiments, the proposed method was evaluated and further compared with two state-of-the-art methods, Global Iterative Closest Point (G-ICP) [7] and Fast Point Feature Histograms (FPFH) [9], respectively. The unregistered 3D point clouds/scans obtained in Exp_1 and Exp_2 were registered independently using these three methods. The evaluation results are presented in the next sections.

### 6.1. Registration Accuracy

**Figure 17:**
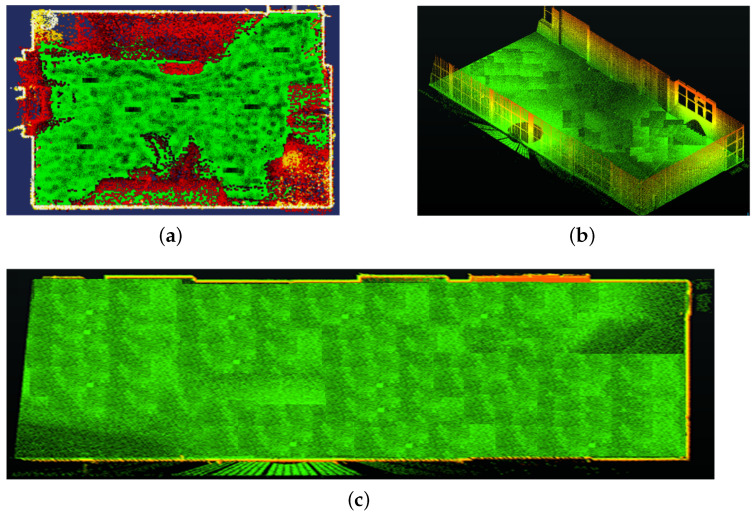
(**a**–**c**) The registration results for the experiments Exp_1 and Exp_2 employing the proposed method.

**Figure 18:**
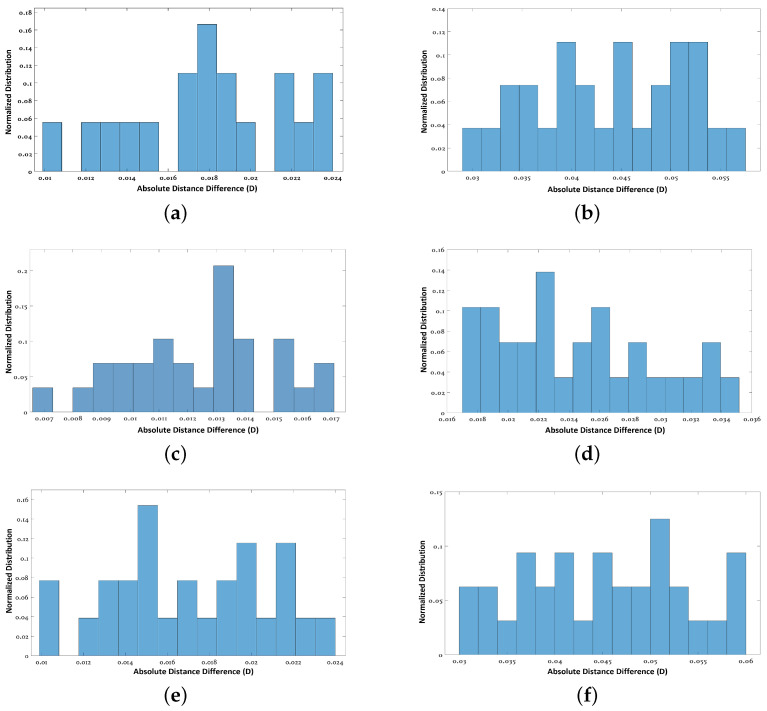
(**a**,**c**,**e**) The absolute distance difference distributions for Exp_1 for the Proposed method, G-ICP [7] and FPFH [9], respectively. (**b**,**d**,**f**) The absolute distance difference distributions for Exp_2 for the three methods, respectively.

The registration results for both experiments are presented in Figure 17. A quantitative analysis is presented in Table 2 and Figure 18 for the two experiments and compared with the state-of-the-art methods [7,9]. ΔE, in the table, presents the registration error calculated in terms of the average absolute difference in dimensions (width and height) of the window and door openings, walls and floor, and compared to the ground truth (similar to [37]); whereas, in Figure 18, absolute distance difference distributions are presented.

The results show that the proposed method is comparable to the other state-of-the-art methods in terms of accuracy.

The registration accuracy of the proposed method was better in Exp_1 and closer to G-ICP as the room size was small and required a smaller number of scans. In the G-ICP algorithm, after successive scan matching, there is a bundle adjustment step at the end to improve the overall scan registration so that the number of scans (or size of acquisition) has less impact on the overall results. Whereas, in the proposed method, the registration error usually tends to accumulate more with a larger number of scans (acquisition size). However, this does not always impact the closed loop error, as seen in the results.

### 6.2. Processing Time

The proposed method along with the other state-of-the-art methods were run on the same laptop (Intel Core i9-10885H processor @2.40 GHz, memory 32 GB (Intel, Santa Clara, CA, USA)) under a Windows 10 operating system and Matlab environment. The results of the registration time (in seconds) are presented in Table 3.

From the results, it can be seen that there is no significant difference in the processing time at this lower scan resolution (number of points per scan).

From the results, it can be seen that there is no significant difference in the processing time at this lower scan resolution (number of points per scan).

As the results presented in Table 3 were inconclusive, Exp_1 was repeated at different scan resolutions by varying the data integration time, i.e., from 0.2s (low resolution) to 0.5s (high resolution). The results are presented in Table 4.

The results clearly show that the strength of the proposed method lies in the computational time as the number of points per scan increases. For methods like G-ICP, the processing time increases manifold with the increase in the number of points as the number of correspondences between points increases. However, the proposed method remains relatively unchanged.

### 6.3. Localization Error

**Figure 19:**
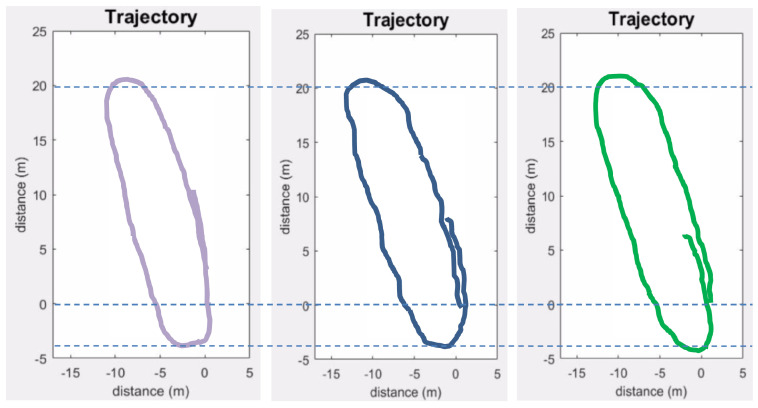
Estimated trajectories (proposed method in blue and odometer/MEMS IMU-based in green) compared to the ground truth (in purple).

The localization error was computed by comparing the trajectory estimated by the proposed method with the predefined trajectory of the mobile robot in Exp_2 (Ground truth). The first scan (at the start point) is considered as the origin or zero position. The method is further compared with the trajectory obtained using an odometer/MEMS IMU-based method [38]. These data were obtained from the odometer and a low-cost IMU embarked on the mobile robot used in Exp_2. The errors of the estimated parameters were calculated at regular intervals of the trajectory in terms of Root-Mean-Square Error (RMSE) values. The results are presented in Figure 19 and Table 5.

The results show that the trajectory estimated by the proposed method contains some abrupt changes resulting in a not-so-smooth (kinky) trajectory, as shown in Figure 19, whereas both the proposed and the odometer/MEMS IMU-based methods suffer from drift.

The proposed method fares slightly better in terms of drift as it employs matching of subsequent scans that automatically corrects some of the errors; however, the overall error tends to accumulate in successive scans. This could be improved by adding some constraints like loop closing.

### 6.4. Improving Localization and Mapping Results

**Figure 20:**
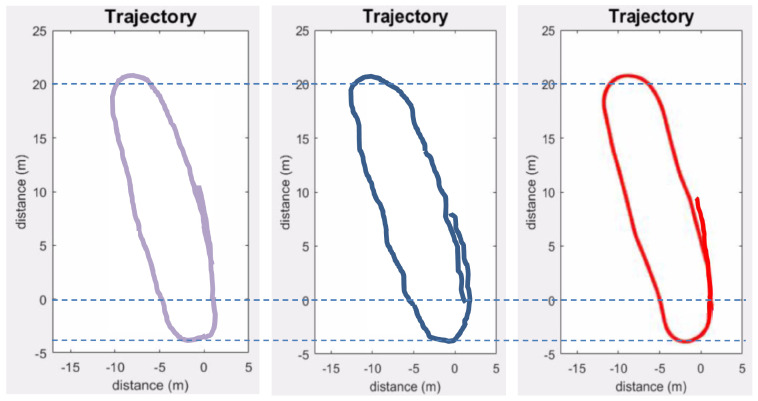
Improved estimated trajectories (without improvement in blue and improved one in red) compared to the ground truth (in purple).

In order to improve upon the localization and mapping results, two different strategies were adopted. The sharp or abrupt changes in the trajectory were improved by employing a curve fitting derivative filter (CFDT), similar to the one proposed in [39], to the successive localization positions. As the proposed method suffers from slight drift in terms of error in the case of larger distance and number of scans, the accuracy of the proposed method was sought to be improved by incorporating some constraints like loop closure. A hard loop closure was employed on the trajectory obtained from the proposed method. The other 3D scans were subsequently adjusted using the CFDT filter to obtain a more accurate and smooth trajectory. The improved localization results from Exp_2 are presented in Figure 20 and Table 6.  

**Figure 21:**
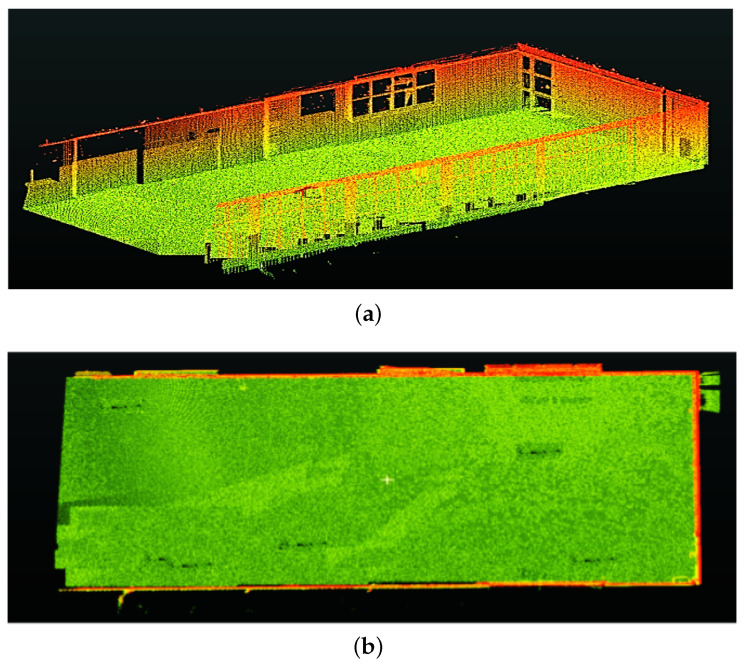
(**a**,**b**) The registration results for Exp_2 after improvements in the proposed method.

The improvement in the localization/trajectory estimation should also improve the overall registration/mapping accuracy. The registration accuracy (Section 6.1) for experiment Exp_2 was recalculated, the improved results are presented in Table 7 and the resulting 3D map (registered point cloud) is shown in Figure 21.

## 7. Conclusions

In this paper, the feasibility of a Spirograph-type non-repetitive scanning pattern LiDAR sensor to achieve robust 3D point cloud registration for localization and mapping applications is presented. First, the comprehensive characterization results of this unique sensor are presented; then, exploiting these results, a new 3D point cloud registration method that exploits the unique scanning pattern of the sensor to register successive 3D scans is proposed. The results evaluated on real data show that the proposed method is comparable with other state-of-the-art methods in terms of accuracy but surpasses them in performance in terms of processing speed as it is shown to be invariant to increase in the number of 3D points per scan. After analysis of the results, a slight drift in error was observed in the case of larger distance, number of scans and also abrupt changes in the trajectory estimation. Both these problems were then successfully addressed by employing a CFDT filter to smooth the trajectory and adding loop closure as a constraint to improve upon the overall localization and registration accuracies by 32.6%.

For future work, it would be considered to perhaps add more constraints along the trajectory, apart from loop closure, to improve the overall accuracy and explore the use of an ICP-like method to further improve or finely register the resulting point cloud at faster rates. In order to make the vehicle/robot navigation more precise and robust, integrating this type of sensor technology with methods like SLAM-SVSF could also be explored. It is also envisaged to further test the proposed method in more complex, cluttered and unstructured environments to improve upon it and expand the use of such a sensor for different field applications. The work presented in this paper represents a promising advancement toward more robust and accurate 3D point cloud registration, with implications for the continued evolution of technologies reliant on LiDAR-based environmental sensing. 

## Figures and Tables

**Table 1:** Main specifications of the MID-70 sensor by LIVOX.

Main Specifications	
Laser Wavelength	905 nm
Detection Range	260m@ 80% reflectivity
Field of View	70° circular
Range Precision	0.02 m
Beam divergence	0.28° (vert.) × 0.03° (horiz.)
Dimension	97 × 64 × 62.7 mm

**Table 2:** Registration accuracy for experiments Exp_1 and Exp_2.

Experiments	Exp_1	Exp_2
	Δ*E* (m)	Δ*E* (m)
Proposed method	0.018	0.043
G-ICP [7]	0.012	0.024
FPFH [9]	0.017	0.044

**Table 3:** Processing time.

Experiments	Exp_1 (s)	Exp_2 (s)
Proposed method	90	900
G-ICP [7]	100	1700
FPFH [9]	95	1295

**Table 4:** Processing time for different scan resolutions.

Integration Time (Resolution)	0.2 s	0.3 s	0.4 s	0.5 s
Proposed method	90	155	205	289
G-ICP [7]	100	250	480	875
FPFH [9]	95	197	352	615

**Table 5:** Estimation of localization errors in terms of RMSE values.

Values	Proposed Method	Odometer/MEMS IMU-Based
Δx (m)	0.026	0.032
Δy (m)	0.021	0.026
θx (deg)	4.1	3.9
θy (deg)	2.7	3.4

**Table 6:** Estimation of localization errors in terms of RMSE values after improvements.

Values	Proposed Method (Without Improvement)	Proposed Method (With Improvement)
Δx (m)	0.026	0.017
Δy (m)	0.021	0.011
θx (deg)	4.1	3.2
θy (deg)	2.7	1.6

**Table 7:** Improvement in registration accuracy for Exp_2.

Improvement in Registration Accuracy	Without Improvement	With (CFDT + Close Loop)	Improvement
	ΔE (m)	ΔE (m)	(%)
Proposed method	0.043	0.029	32.6

## Data Availability

Data sharing is not applicable to this article.
